# Analysis of influencing factors of HPV vaccination willingness of female sex workers in urban entertainment venues based on the IMB model in Guangxi, China

**DOI:** 10.1186/s12905-024-02962-y

**Published:** 2024-02-24

**Authors:** Zhi-yan He, Jun-hong Wei, Jian-ming Jiang, Rui Hu, Guang-zi Qi

**Affiliations:** 1 College of Public Health and Management, Youjiang Medical University for Nationalities, Baise, 533000 Guangxi China; 2 Modern Industrial College of Biomedicine and Great Health, Youjiang Medical University for Nationalities, Baise, 533000 Guangxi China; 3 Key Laboratory of Research on Environment and Population Health in aluminium mining areas (Youjiang Medical University for Nationalities), Education Department of Guangxi Zhuang Autonomous Region, Baise, 533000 Guangxi China; 4 Key Laboratory of Research on Environmental pollution and health risk assessment, Youjiang Medical University for Nationalities, Baise, 533000 Guangxi China; 5 College of Basic Medical Sciences, Youjiang Medical University for Nationalities, Baise, 533000 Guangxi China

**Keywords:** Human papilloma virus vaccine, Inoculation willingness, Information-motivation-behavior model, Entertainment venue, Female sex worker, Logistic regression analysis

## Abstract

**Objective:**

Understanding HPV vaccination willingness and its influencing factors among female sex workers (FSWs) in entertainment venues in an urban area of Guangxi, China.

**Methods:**

From 15 August to 15 October 2022, FSWs in entertainment venues with commercial sex trade in an urban area of Guangxi were selected as the study subjects for the questionnaire survey using the method of intentional sampling. The questionnaire based on the information-motivation-behavior (IMB) skills model was used to collect the basic characteristics, HPV and HPV vaccine-related information and cognition, motivation to vaccinate, behavioral skills and willingness to vaccinate from the research targets. A multifactor logistic regression model was used to analyze the factors influencing the research targets' willingness to receive HPV vaccination.

**Results:**

Of the 921 research targets, 712 (77.31%) were willing to receive HPV vaccination. The higher the level of knowledge regarding HPV and HPV vaccine-related information, the higher the motivation for HPV vaccination. In addition, the higher the behavioral skills score, the higher the willingness of FSWs in entertainment venues to receive HPV vaccination (*P*<0.001). FSWs in entertainment venues with lower venue grades [*OR(95% CI)*=0.693 (0.539, 0.891), *P*=0.004] were more reluctant to receive HPV vaccination. Those who favored the effectiveness of the vaccine in preventing the disease [*OR(95% CI)*=2.144 (1.449, 3.174), *P*<0.001] and those who had heard of HPV vaccine [*OR(95% CI)*=2.105 (1.451, 3.054), *P*<0.001], were able to perceive the benefits of HPV vaccination [*OR(95% CI)*=1.134 (1.045, 1.230), *P*=0.002]. These individuals acquired greater behavioral skills i.e., self-decision making for HPV vaccination [*OR(95% CI)*=1.130 (1.008, 1.267), *P*=0.036] and self-efficacy [*OR(95% CI)*=1.135 (1.081, 1.191), *P*<0.001] and they were more willing to receive HPV vaccine.

**Conclusions:**

There was a relatively high HPV vaccination willingness among FSWs in entertainment venues in an urban area of Guangxi, China. Attention should be focused on introducing the benefits of primary prevention measures such as the HPV vaccine for individuals and behavioral skills for HPV vaccination in order to increase their willingness to be vaccinated thus increasing their HPV vaccination rate.

Cervical cancer (CC) is one of the main malignant tumor that threatens women's health, with the second highest incidence and mortality rate from cancers affecting females globally and it is the highest in developing countries [1]. CC has the second highest incidence and third highest mortality rate of malignant tumors among women in the 15-44 age group in China [2]. In recent years, the incidence and mortality rates from CC in developing countries have been on the rise and it tends to occur in the younger age groups [3, 4]. The development of CC is the result of a multifactorial, multigenic and multistep process [5]. Persistent human papilloma virus (HPV) infection is the main cause of CC and its precancerous lesions [6, 7]. HPV infection is common in the population and it has various routes of infection, but occurs mainly through sexual transmission. Epidemiological studies have shown that HPV infection is related to a variety of factors, and the high-risk factors include early age of sexual initiation, unclean sex, multiple sexual partners, excessive alcohol consumption, smoking and other bad habits. In particular, sexual disorders is one of the most important factors leading to HPV infection in women [8–10].

CC is the only malignant tumor that can be prevented by vaccination and this would lead to a reduction in mortality rate. As a primary preventive measure against CC, the HPV vaccine can prevent 70 to 90% of HPV-associated cancers, and the progression of HPV infection, cervical carcinogenesis and treatment are also closely related as to whether or not to receive the HPV vaccine [11, 12]. Therefore, the incidence of CC can be reduced through effective measures such as raising women's awareness of HPV and HPV vaccine and early vaccination against HPV [13, 14]. According to the statistics, the vaccination rate for women aged 9 to 45 years in China in 2017-2020 was only about 3% [15]. There is currently little information on HPV-related knowledge and attitudes among adult females in the Asia-Pacific region, particularly among high-risk groups such as female sex workers (FSWs) in entertainment venues [16, 17]. FSWs in entertainment venues are a high-risk group for HPV infection due to their complex occupations and living environments, relatively open sexual attitudes and weak awareness of their own health [18]. Understanding the HPV vaccination willingness of women in this occupation and its influencing factors is of great significance for the prevention and control of HPV infection as well as the development and progression of CC in China.

The information-motivation-behavior (IMB) skill model was first proposed by JD Fisher and WA Fisher in 1992. As a new behavioral intervention theory, the IMB model has been increasingly used in public health [19–22]. The promotion of HPV vaccination as a health behavior is influenced by information, motivation and behavioral skills in line with the IMB model [23, 24]. Based on the IMB skill model, this study investigated the current status of FSWs' knowledge of HPV and HPV vaccine in entertainment venues in an urban area of Guangxi, China, and analyzed their willingness to be vaccinated against HPV and its influencing factors. This will provide a theoretical basis for the development of health education and promotional measures on CC, HPV and HPV vaccine by disease prevention agencies and women's healthcare medical units.

## Subjects and methods

### Research targets

The target population of this survey was FSWs in entertainment venues with a commercial sex trade in an urban area of Guangxi, and the survey period of study was from 15 August to 15 October 2022. Referring to the classification of "Places where high-risk behavior occur in clandestine prostitution" in the Operation Manual of the National AIDS Sentinel Surveillance Implementation Plan (Trial), and combining this with the actual situation in an urban area of Guangxi, entertainment venues were stratified according to high, medium and low grades. The high-end included nightclubs, entertainment clubs, KTV, dance halls, bars, saunas and bathing centers; the mid-range included health care massage shops, foot bath shops and hotels; the low-end included hair salons, rental houses, roadside shops, small restaurants, streets and parks.

### Investigative tools

Drawing on references within the domestic and international literature [23, 25–28], we developed a questionnaire on the cognition, attitude, psychological behavior towards the human papilloma virus (HPV) vaccine. The content included: a basic information questionnaire, a questionnaire based on the IMB model of HPV and HPV vaccine-related information and cognition, vaccination motivation and behavioral skills, and a questionnaire on the willingness to be vaccinated against HPV.

#### Basic information

This included general demographic characteristics of the study population, sexuality-related content and health-related lifestyle habits.

#### IMB Module

The IMB model questionnaire has 45 entries in 3 dimensions, of which the HPV- and HPV vaccine-related information questions include 5 entries on basic understanding and 12 entries on related knowledge. The related knowledge refers to judgement questions with three options: "right", "wrong" and "not sure", and the scoring is 1, 0 and 0, respectively, for these. The total score ranges from 0 to 12, with higher scores indicating a higher level of knowledge regarding CC, HPV and HPV vaccine among the research targets. The HPV and HPV vaccination motivation questionnaire was divided into personal motivation, i.e., perceived severity, perceived susceptibility, perceived benefits, perceived barriers and social motivation, with a total of 20 entries. This was scored on a 5-point Likert scale, with a score of "1=Strongly Disapprove", "2 = Disapprove", "3 = Unsure", "4 = Approve" and "5 = Strongly Approve". Six of the entries were reverse scored, "I am worried about the side effects of the HPV vaccine" "I am afraid of unbearable needle pain from the HPV vaccine" "I am hesitant to get the HPV vaccine" "I am worried that it is difficult to afford the cost of HPV vaccination" "I am worried that the HPV vaccination procedure is complicated and a waste of time". For these the higher total scores indicated stronger attitudes towards HPV vaccination among the research targets. Behavioral skills for HPV and HPV vaccination included self-decision making and self-efficacy, with a total of eight entries, all rated on a Likert scale of 1-5, with scores ranging from "1=Strongly Disapprove", "2=Disapprove", "3=Unsure", "4=Approve", and "5=Strongly Approve". The higher scores indicated a stronger psychological behavior towards HPV vaccination among the research targets. The total *Cronbach's α* coefficient for the scale dimensions of this questionnaire was 0.828.

#### Willingness to be vaccinated with the HPV vaccine

That is, whether the research targets themselves were willing to be vaccinated against HPV.

### Survey methodology and quality control

The cross-sectional survey used a purposive sampling method to select the study subjects. Through the outreach intervention staff of the Centre for Disease Control and Prevention in an urban area of Guangxi, we contacted FSWs via the owners of entertainment venues in advance for support and assistance. After obtaining consent, the FSWs entered the survey site according to the agreed-upon time for the recruitment of study subjects. Informed consent was obtained from the research subjects before the survey, and the principle of complete voluntariness was adopted, with the respondents having the right to withdraw and refuse to participate at any time. The research subjects were not forced to go against their will, and the confidentiality of the situation of the respondents participating in the study was strictly maintained. On-site face-to-face anonymous questionnaire surveys were conducted by uniformly trained investigators, with each respondent participating only once to avoid repetition and crossover. In order to ensure the validity and credibility of the questionnaire, all the scales used were issued and recovered by the researchers on the spot. In addition, the purpose of the questionnaire and the matters requiring attention were filled in patiently and in detail while it was issued to the participant, so as to avoid, as far as possible, any survey errors caused by the researcher during the process of collecting the answered questions. When the questionnaires were collected, their completion was carefully examined, and if they were incomplete, doubtful or contradictory, these problems were addressed with the respondents in a timely manner and supplemented with corrections. Any questionnaires that did not meet the requirements were excluded. A total of 923 FSWs in entertainment venues participated in filling out the 2 non-autonomous questionnaires. A total of 921 valid questionnaires were recovered, and the recovery rate was 99.78%. This number was the sample size for this investigation and the number of ethical proofs obtained was 921.

### Statistical methods and statistical software

#### Statistical methods

In this study, firstly, the statistical description of the measurement data was performed using the means ± standard deviations $\left(\overline{\chi }\pm s\right)$, and the statistical description of the count data was by using frequencies (%). The chi-square and *t* tests were used to compare the differences between study subjects with different vaccination intentions.

Logistic regression is a probabilistic model for generalized linear regression analysis, which is a machine learning method for solving dichotomous (0 or 1) problems, estimating the likelihood of a parameter, and it is commonly used to find the risk factors for a particular disease. It can be used to predict the probability of a particular disease or situation occurring in the presence of different independent variables, or judging the probability that someone belongs to a certain disease group or belongs to a certain situation. It is widely used in regression models where the dependent variable is a dichotomous categorical variable [29–32]. The outcome of the dependent variable, *Y,* for the willingness of FSWs in entertainment venues to receive HPV vaccination analyzed in this study was divided into willingness and unwillingness to participate, which is a dichotomous categorical variable that can be analyzed using a binary logistic regression model. In this study, we used multifactorial logistic regression model to analyze the influencing factors of HPV vaccination willingness within the study population. The criteria for variable entry and removal were ɑ_entry_ = 0.05 and ɑ_removal_ = 0.10. The difference was considered to be statistically significant when *P*<0.05.

#### Statistical software

In this study, EpiData 3.1 software was used to establish the database, the data were exported and collated by Excel 2019 software, and the data were descriptively analyzed using SPSS 26.0 software.

## Results

### Basic information

Of the 921 research targets, 378 (41.04%) were in high-grade recreational establishments, 227 (24.65%) were in mid-range, and 316 (34.31%) were in low-grade ones. The ages of the research targets were concentrated in the age group of 27 to 45 years old (476, 51.68%). The ethnic groups were mainly Han (398, 43.21%) and Zhuang (388, 42.13%). The education level was mostly high school/vocational high school/technical school/technical secondary school (371, 40.28%). Five hundred one people (54.40%) had a family location in rural areas and 566 subjects (61.45%) had a monthly personal income ≥ 3800 yuan. The length of time in their current occupation was mostly >12 months (496, 53.85%). The highest educational attainment of their fathers (403, 43.76%) and mothers (364, 39.52%) was concentrated in junior high school. A total of 437 (47.45%) were unmarried, of whom 207 (22.48%) had no boyfriend, 230 (24.97%) had a boyfriend and 360 (39.09%) were married. The age of first sexual intercourse was mostly less than 20 years old (512, 55.59%), and condoms were mostly used during sexual intercourse (503, 54.61%) as contraceptive measures, and by far the majority of those had ≥3 sexual partners (357, 38.76%); 534 (57.98%) said that premarital sex was acceptable. Four hundred eighty-one (52.23%) have received sex education (sexual health training, male and female reproductive system learning), and 494 (53.64%) have received training on infectious diseases, preventive medicine education and sexually transmitted diseases such as AIDS. 256 subjects (27.80%) said some of their relatives, friends or other close relationships had cancer (including but not limited to CC). Only 333 people (36.16%) had neither a tobacco or alcohol addiction (Table 1).

**Table 1:** The basic characteristics of female sex workers in different entertainment venues and their willingness to participate in HPV vaccination

Variables	Total(%)	Willing to be vaccinated(%)	Reluctance to be vaccinated(%)	*χ*^*2*^	*P*
Number of people	921(100.00)	712(77.31)	209(22.69)		
Class of venue				134.138	<0.001
High -grade	378(41.04)	359(94.97)	19(5.03)		
Mid-range	227(24.65)	126(55.51)	101(44.49)		
Low-grade	316(34.31)	227(71.84)	89(28.16)		
Age (weeks)				31.052	<0.001
<20	87(9.45)	65(74.71)	22(25.29)		
20~26	282(30.62)	199(70.57)	83(29.43)		
27~45	476(51.68)	401(84.24)	75(15.76)		
≥46	76(8.25)	47(61.84)	29(38.16)		
Ethnicity				9.551	0.008
Han Chinese	398(43.21)	303(76.13)	95(23.87)		
Zhuang	388(42.13)	316(81.44)	72(18.56)		
Other Minorities	135(14.66)	93(68.89)	42(31.11)		
Education level				9.258	0.055
Elementary school and below	52(5.65)	40(76.92)	12(23.08)		
Junior High School	304(33.01)	239(78.62)	65(21.38)		
High school or vocational high school or technical school or junior high school	371(40.28)	297(80.05)	74(19.95)		
College	132(14.33)	96(72.73)	36(27.27)		
Bachelor's degree or above	62(6.73)	40(64.52)	22(35.48)		
Home Location				0.549	0.459
City	420(45.60)	320(76.19)	100(23.81)		
Rural	501(54.40)	392(78.24)	109(21.76)		
Personal monthly income (Yuan per month)				20.336	<0.001
<1500	88(9.55)	64(72.73)	24(27.27)		
1500~	267(28.99)	183(68.54)	84(31.46)		
≥3800	566(61.45)	465(82.16)	101(17.84)		
Length of time in current occupation (months)				17.031	0.001
<1	50(5.43)	39(78.00)	11(22.00)		
1~6	168(18.24)	122(72.62)	46(27.38)		
7~12	207(22.48)	143(69.08)	64(30.92)		
>12	496(53.85)	408(82.26)	88(17.74)		
The highest level of education of the father				14.130	0.007
Elementary school and below	260(28.23)	206(79.23)	54(20.77)		
Junior High School	403(43.76)	327(81.14)	76(18.86)		
High school or vocational high school or technical school or junior high school	183(19.87)	127(69.40)	56(30.60)		
College	57(6.19)	38(66.67)	19(33.33)		
Bachelor's degree or above	18(1.95)	14(77.78)	4(22.22)		
The highest level of education of the mother				13.146	0.011
Elementary school and below	332(36.05)	258(77.71)	74(22.29)		
Junior High School	364(39.52)	295(81.04)	69(18.96)		
High school or vocational high school or technical school or junior high school	178(19.33)	130(73.03)	48(26.97)		
College	29(3.15)	16(55.17)	13(44.83)		
Bachelor's degree or above	18(1.95)	13(72.22)	5(27.78)		
Marital Status				6.568	0.087
Unmarried, no boyfriend	207(22.48)	164(79.23)	43(20.77)		
Unmarried, have a boyfriend	230(24.97)	178(77.39)	52(22.61)		
Married	360(39.09)	285(79.17)	75(20.83)		
Divorced or widowed	124(13.46)	85(68.55)	39(31.45)		
Age of first sexual intercourse				4.791	0.091
Never had sexual intercourse	52(5.65)	34(65.38)	18(34.62)		
<20 years old	512(55.59)	396(77.34)	116(22.66)		
≥20 years old	357(38.76)	282(78.99)	75(21.01)		
Contraceptive measures used during sexual intercourse				70.485	<0.001
Never had sexual intercourse	52(5.65)	34(65.38)	18(34.62)		
Condoms	503(54.61)	440(87.48)	63(12.52)		
Intrauterine device (IUD)	112(12.16)	81(72.32)	31(27.68)		
Contraceptives	104(11.29)	66(63.46)	38(36.54)		
No contraception	150(16.29)	91(60.67)	59(39.33)		
Number of sexual partners to date				4.817	0.186
0	52(5.65)	34(65.38)	18(34.62)		
1	284(30.84)	221(77.82)	63(22.18)		
2	228(24.76)	181(79.39)	47(20.61)		
≥3	357(38.76)	276(77.31)	81(22.69)		
Attitudes toward premarital sex				35.158	<0.001
Agree or support	122(13.25)	88(72.13)	34(27.87)		
Acceptable	534(57.98)	449(84.08)	85(15.92)		
Oppose or reject	77(8.36)	52(67.53)	25(32.47)		
Does not matter	188(20.41)	123(65.43)	65(34.57)		
Whether or not they have received sex education (sexual health training, male and female reproductive system learning)				11.098	0.001
No	440(47.77)	319(72.50)	121(27.50)		
Yes	481(52.23)	393(81.70)	88(18.30)		
Whether they have received training on infectious diseases, preventive medicine education or knowledge about sexually transmitted diseases such as AIDS				2.062	0.151
No	427(46.36)	321(75.18)	106(24.82)		
Yes	494(53.64)	391(79.15)	103(20.85)		
Any family member, friend or other close relative who has cancer (including but not limited to cervical cancer)				5.102	0.078
No	409(44.41)	328(80.20)	81(19.80)		
Yes	256(27.80)	198(77.34)	58(22.66)		
Not sure	256(27.80)	186(72.66)	70(27.34)		
Tobacco and alcohol hobbies				21.408	<0.001
None	333(36.16)	269(80.78)	64(19.22)		
Smoking	60(6.51)	39(65.00)	21(35.00)		
Drinking	233(25.30)	196(84.12)	37(15.88)		
Smoking and drinking	295(32.03)	208(70.51)	87(29.49)		

### HPV vaccination intention of FSWs in different basic situations entertainment places

Seven hundred twelve (77.31%) of the study population were willing to be vaccinated against HPV. The class of the establishment, age, ethnicity, monthly personal income, length of time in current occupation, father's and mother's highest levels of education, contraception used at the time of sexual intercourse, attitude towards premarital sex, whether or not they had received sex education, tobacco and alcohol addiction differed between FSWs in entertainment establishments who were willing and unwilling to receive HPV vaccination (*P*<0.05 in all parameters) in entertainment venues (Table 1).

### The IMB model dimension scores and HPV vaccination intention among FSWs in entertainment venues

FSWs in entertainment venues who were willing to receive HPV vaccination favoured the vaccine's effectiveness in preventing disease, had significantly higher knowledge of HPV, HPV-related diseases, HPV vaccine, and had actively learned about the HPV vaccine than those who were reluctant to receive the vaccine, and had higher scores for knowledge about HPV and HPV vaccine-related knowledge than those who were reluctant to receive the vaccine (*P*<0.001).Perceived severity and susceptibility to HPV, perceived benefits of HPV vaccination, social motivation, and behavioral skills related to HPV vaccination, i.e., self-decision making and self-efficacy, were higher among FSWs in entertainment venues who were willing to vaccinate than those who were unwilling to vaccinate, and the difference was statistically significant (*P*< 0.05) (Table 2).

**Table 2:** Comparison of IMB model dimension scores and HPV vaccination intention among female sex workers in entertainment venues

IMB Module Variables	Willing to be vaccinated (%)	Reluctance to be vaccinated (%)	t-value/*χ*^*2*^	*P*
Information				
Do you agree that vaccines are effective in preventing disease?			101.825	<0.001
Yes	615	113		
No	97	96		
Have you heard of HPV?			69.647	<0.001
Yes	553	100		
No	159	109		
Have you heard of HPV-related diseases?			38.670	<0.001
Yes	503	99		
No	209	110		
Have you heard of the HPV vaccine?			85.370	<0.001
Yes	551	92		
No	161	117		
Have you proactively learned about the HPV vaccine?			41.699	<0.001
Yes	409	67		
No	303	142		
HPV and HPV vaccine knowledge score	5.36±2.73	4.20±2.09	6.566	<0.001
Motivation				
Perceived severity	15.79±3.28	14.05±3.87	5.899	<0.001
Perceived susceptibility	6.04±1.93	5.64±1.83	2.649	0.008
Perceived benefits	11.99±2.30	9.97±2.55	10.320	<0.001
Perceptual hindrance	17.27±4.45	17.11±4.30	0.469	0.639
Social motivation	16.77±3.21	15.34±3.67	5.081	<0.001
Behavioral Skills				
Self-determination	8.49±1.43	7.41±1.81	7.895	<0.001
Self-efficacy	22.83±4.49	18.46±4.17	12.536	<0.001

### Multifactor analysis of HPV vaccination intentions among FSWs in entertainment venues

HPV vaccination willingness was used as the dependent variable (willingness = 1, unwillingness = 0), and variables with statistically significant differences (*P*<0.05) in the above univariate analyses were used as independent variables, the *enter* method was used to establish a multi-factor logistic regression model, and the independent variables and the results are shown in Table 3.

**Table 3:** Multi-factor logistic regression analysis of HPV vaccination willingness of female sex workers in entertainment venues

Variables and classification	Assignment situation	*β*	Standard error	Wald *χ*^*2*^	*P*	OR(95% CI)
Basic information						
Class of venue	Grade information (from highest to lowest)	-0.335	0.148	5.138	0.023	0.716(0.536~0.956)
Age	Grade information (from lowest to highest)	-0.003	0.123	0.001	0.980	0.997(0.784~1.268)
Ethnicity						
Han Chinese	Control group					1.000
Zhuang		0.286	0.213	1.805	0.179	1.331(0.877~2.021)
Other Minorities		0.120	0.267	0.202	0.653	1.127(0.668~1.903)
Personal monthly income (Yuan per month)	Grade information (from the lowest to the highest)	-0.118	0.167	0.501	0.479	0.889(0.641~1.232)
Length of time in current occupation	Grade information (from the lowest to the highest)	0.074	0.116	0.400	0.527	1.076(0.857~1.352)
The highest level of education of the father	Grade information (from the lowest to the highest)	-0.032	0.134	0.056	0.814	0.969(0.745~1.260)
Highest level of education of the mother	Grade information (from the lowest to the highest)	0.014	0.136	0.010	0.920	1.014(0.777~1.322)
Contraceptive measures used during sexual intercourse						
Never had sexual intercourse		-0.042	0.425	0.010	0.921	0.959(0.417~2.206)
Take contraceptive measures such as condoms, IUDs, and birth control pills		0.333	0.240	1.927	0.165	1.395(0.872~2.232)
No contraception	Control group					1.000
Attitudes toward premarital sex	Agree or support, acceptable, indifferent	0.506	0.319	2.523	0.112	1.659(0.888~3.097)
	Oppose or reject					1.000
Whether or not they have received sex education (sexual health training, male and female reproductive system studies)	No(Control group)					1.000
	Yes	-0.028	0.199	0.020	0.888	0.973(0.659~1.435)
Tobacco and alcohol hobbies	No					1.000
	Smoking, drinking alcohol or smoking and drinking alcohol	-0.428	0.210	4.137	0.042	0.652(0.432~0.985)
IMB Module Variables						
Information						
Do you agree that vaccines are effective in preventing disease?	No(Control group)					1.000
	Yes	0.694	0.213	10.602	0.001	2.001(1.318~3.039)
Have you heard of HPV?	No(Control group)					1.000
	Yes	0.285	0.221	1.659	0.198	1.329(0.862~2.050)
Have you heard of HPV-related diseases?	No(Control group)					1.000
	Yes	-0.064	0.220	0.085	0.771	0.938(0.609~1.444)
Have you heard of the HPV vaccine?	No(Control group)					1.000
	Yes	0.633	0.218	8.475	0.004	1.884(1.230~2.886)
Have you proactively learned about the HPV vaccine?	No(Control group)					1.000
	Yes	0.032	0.228	0.020	0.888	1.033(0.661~1.613)
HPV and HPV vaccine knowledge score	Continuous variables	-0.019	0.045	0.188	0.664	0.981(0.899~1.070)
Motivation						
Perceived severity	Continuous variables	0.009	0.029	0.095	0.758	1.009(0.954~1.067)
Perceived susceptibility	Continuous variables	0.032	0.055	0.350	0.554	1.033(0.928~1.150)
Perceived benefits	Continuous variables	0.130	0.044	8.564	0.003	1.139(1.044~1.243)
Social motivation	Continuous variables	0.007	0.031	0.049	0.825	1.007(0.948~1.069)
Behavioral Skills						
Self-determination	Continuous variables	0.125	0.062	4.094	0.043	1.134(1.004~1.280)
Self-efficacy	Continuous variables	0.115	0.026	18.991	<0.001	1.121(1.065~1.181)

### Validity, fitting effect and predictive value of the logistic regression model

A logistic regression model likelihood ratio *χ*^2^=246.338, *DF*=25, *P*<0.001 were obtained, indicating that there was statistical significance in the model building in this study. *Waldχ*^2^=242.754, *DF*=1, *P*<0.001, indicated that the model construction was valid. The Hosmer-Lemeshow goodness-of-fit test produced *Chi-Square*=14.360, *DF*=8, *P*=0.073>0.05, indicating that the model was well fitted and that the model was able to correctly classify 80.2% of the study population. The results of the goodness-of-fit and likelihood ratio tests of this multifactor Logistic regression model showed that the model's goodness-of-fit was *-2logL*=740.121, which inferred that the value of its likelihood function was smaller than either of the likelihood functions in the one-factor logistic regression. However, the results of analysis in Table 3 showed that at *ɑ*=0.05, only the class of venue (*X*_*1*_), smoking and drinking habits (*X*_*2*_), whether they agreed that the vaccine was effective in preventing the disease (*X*_*3*_), whether they had heard of the HPV vaccine (*X*_*4*_), perceived benefits of HPV vaccination (*X*_*5*_), mastering the regression coefficients of more behavioral skills for HPV vaccination, namely self-decision-making (*X*_*6*_) and self-efficacy (*X*_*7*_), were estimated to be statistically significant.

The influencing factors that were not statistically significant were removed and the data was re-fitted, as shown in Table 4. The results showed that the willingness of FSWs in entertainment venues to be vaccinated against HPV was associated with the class of the venue, tobacco and alcohol addiction, whether they were in favor of the vaccine being effective in preventing disease, whether they had heard of HPV vaccination, perceived benefits of HPV vaccination and the acquisition of more behavioral skills for HPV vaccination, i.e., self-decision making and self-efficacy. Among them, FSWs in lower grade entertainment venues were less willing to receive the HPV vaccine [*OR(95% CI)*=0.693(0.539, 0.891),*P*=0.004]. Those who agreed that vaccines can effectively prevent diseases were more likely to be vaccinated [*OR(95% CI)*=2.144 (1.449, 3.174), *P*<0.001]. Those who had heard about the HPV vaccine [*OR(95% CI)*=2.105 (1.451, 3.054), *P*<0.001] and the ones who could perceive the benefits of HPV vaccination [*OR(95% CI)*=1.134 (1.045, 1.230), *P*=0.002] were both more likely to get the HPV vaccine. The subjects with more behavioral skills for HPV vaccination i.e., self-decision [*OR(95% CI)*=1.130 (1.008, 1.267), *P*=0.036] and self-efficacy [*OR(95% CI)*=1.135 (1.081, 1.191), *P*<0.001] were also more willing to receive HPV vaccination. The likelihood ratio of the multifactorial logistic regression model obtained by refitting *χ*^2^=234.692, *DF*=7, *P*<0.001, was statistically significant, indicating that the model was meaningful on these points. The Hosmer-Lemeshow goodness-of-fit test pointed out that *chi-square*=13.769, *DF*=8, *P*=0.088>0.05, indicating that the model fitting effect was good. In addition, the goodness-of-fit of the model was *-2logL*=751.767, at which point the model is able to correctly classify 80.9% of the respondents. By using the statistics and analytical data of the logistic regression model at this time, the predicted probability *P* of FSWs in entertainment venues willing to receive the HPV vaccine was obtained. The cumulative superiority logistic regression model was: *Logit(P)* = -3.710-0.367*X*_*1*_-0.358*X*_*2*_+0.763*X*_*3*_+0.744*X*_*4*_+0.126*X*_*5*_+ 0.123*X*_*6*_+0.126*X*_*7*_.

**Table 4:**
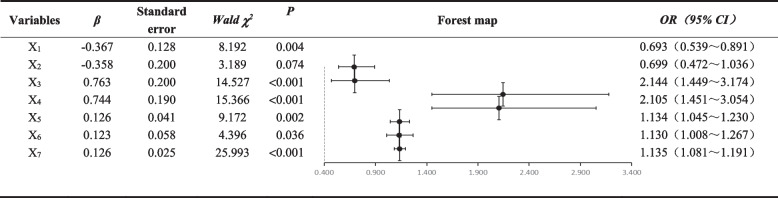
Results of the re-fitted multi-factor logistic regression analysis

## Discussion

The 921 subjects in this study were aged between 27 and 45 years old and were at the peak of their sexual desire in young adulthood [33]. Their education level was mostly up to high, vocational high, technical and secondary schooling and their family locations were mostly in rural areas. Their parents' highest education level was concentrated in junior high school, their workplaces were mostly in high-grade and their monthly income is mostly more than or equal 3,800 RMB. The length of time they had been engaged in their current occupation was more than 12 months, and they considered that they may had chosen to enter the society earlier due to the constraints of their education environment or the family's conditions. They may have chosen to engage in this occupation due to the fact that they were unable to find a high-paying job because of their personal knowledge level and technical ability. It is suggested that the local government should pay more attention to rural young adult women and increase the opportunities for them to learn knowledge and skills through education. This would improve their education level and employability and allow them to obtain a property through legal and compliant ways. These measures are likely to reduce the number of FSWs in entertainment venues at the source, so as to fundamentally curb the occurrence and development of HPV and other diseases that are mainly transmitted through the sexual route.

The majority of the research subjects were unmarried, and most of them indicated that premarital sex was acceptable. Those who had had sex were mostly younger than 20 years old at the time of their first sexual intercourse, and the number of sexual partners was mostly more than or equal 3, and only about one-third of them had neither tobacco nor alcohol habits, which is consistent with the high-risk factors for HPV infection [34, 35]. This strongly suggests that women in this occupation are a high-risk group for HPV infection. It has been shown that single HPV infection increases the risk of CC by 19.9-fold, whereas multiple HPV infections increase this risk by 31.8-fold [36], and the strongest predictors of infection with multiple HPV types were a young age and multiple sexual partners [37]. In addition, multiple HPV infections may also have a synergistic effect on cervical lesions, thus affecting the prognosis of CC [38, 39]. This suggests that the risks of multiple HPV infections and CC are much higher in the study population than in females generally, and that the progression of the disease and poor prognosis may be more severe in the former. Most FSWs in entertainment venues use condoms as a contraceptive measure. Even though condoms can reduce the risk of HPV infection to a certain extent, they are not absolutely avoidable, so a multi-pronged approach towards HPV vaccination, condom wearing and regular screening is still advocated for the prevention of HPV infection and CC.

Only 52.23 and 53.64% of the study subjects had received sex education (sexual health training, male and female reproductive system study) and infectious disease and preventive medicine education or training on sexually transmitted diseases such as AIDS. This indicated that the level of understanding of this occupational group of women in terms of gender education and sexually transmitted diseases is not high, and that they were unable to protect their personal health from being infringed upon on their own. Therefore, the community, women's federations and other organizations should pay attention to the management of the group of women with this occupation. Hence these organizations should pay more attention to the management of such working women, strengthen their education and popularize the knowledge regarding sexually transmitted diseases. They should also call and encourage them to undergo regular medical check-ups, pay attention to the two types of cancer screening, and raise their levels of knowledge and awareness about healthcare.

China is currently in the primary stage of HPV vaccine promotion and research and development, and FSWs in entertainment venues, as a high-risk group for HPV infection, are a key population of concern for HPV vaccination. In addition, improving women's knowledge of HPV and HPV vaccination rates in this population is crucial to reducing the incidence of CC and protecting women's health in China. The results of this study showed that the HPV vaccination willingness of FSWs in entertainment venues in an urban area of Guangxi, China, was 77.31%, which was higher than the average level in China (62.2%) [40] but lower than those in Peru (97.5%) [41] and Phnom Penh and Cambodia (100%) [16]. The approval of HPV vaccine in China was recent and it has been only been on the market for a short period of time, and the dissemination rate and popularity of HPV-related knowledge and HPV vaccination information are not high, and the public's understanding of it is even less optimistic [42]. In recent years, an urban area in Guangxi, China, has strengthened the detection and supervision of sexually transmitted diseases among FSWs in entertainment venues, and the publicity and popularization of HPV and HPV vaccine have been significantly improved, resulting in a relatively high willingness to vaccinate FSWs in these establishments in this region.

Some studies have shown that the factors affecting HPV vaccination include personal factors as well as social, family and interpersonal relationships [24]. Based on the theory of the IMB model, the present investigation analyzed the factors influencing HPV vaccination intentions of FSWs in an urban entertainment area in Guangxi, China, in terms of information, motivation and behavioral skills.

### Information factors

This study found that the higher the level of knowledge about CC, HPV and HPV vaccine, the higher was the willingness to receive the HPV vaccine among FSWs in entertainment venues. The willingness of those who agreed that the vaccine could effectively prevent diseases was 2.144 times higher than that of those who did not agree with the HPV vaccine. The reason may be that they are more willing to receive the HPV vaccine to reduce the probability of HPV infection and CC because of the benefits to their health and daily life due to the reduction in the chances of contracting the disease or the reduction in the symptoms of the disease in the course of the previous preventive vaccination. They also recognize the effectiveness of the vaccine as a primary preventive measure. FSWs in entertainment venues who have heard of the HPV vaccine were more willing to receive it, which is 2.105 times higher than those who had not heard of the vaccine. It was only when they had heard that the HPV vaccine could have a preventive effect on HPV infection and CC, did they choose to selectively receive the HPV vaccine according to their own situation. It is suggested that communities, women's associations and disease prevention and control agencies should continue to strengthen the dissemination of information regarding the HPV vaccine in their publicity campaigns, so that more people of the appropriate age and key populations have the means to learn about it. At the same time, they should innovate publicity channels, such as through social media platforms or the development of peer education liaisons, so as to enhance women's knowledge of CC, HPV and HPV vaccine, and to increase their willingness to be vaccinated thus increasing the vaccination rate.

### Motivational factors

The stronger the motivation of the study population for HPV and HPV vaccination, the higher the willingness to receive HPV vaccination. Among the perceived benefits was one of the main reasons influencing the willingness of FSWs in entertainment venues to be vaccinated against HPV, which is different from the findings of other populations such as female university students in China [24, 43]. The reason may be that FSWs in entertainment venues were able to perceive the serious impact of HPV infection and CC on themselves and their occupations due to the special nature of their work, and to a certain extent they understand the susceptibility of this to HPV infection. Therefore, they were able to overcome the impediments to HPV vaccination such as vaccine safety, efficacy and side effects, and meanwhile they have a certain economic basis and self-determination ability compared with university students. The results showed that those who could perceive the benefits of HPV vaccination were more willing to receive the HPV vaccine, which is 1.134 times higher than those who could not comprehend the benefits. This coincided with the fact that those who agreed that vaccines can be effective in preventing diseases were more willing to receive them, and suggested that vaccine production and development institutions should pay attention to the effectiveness of the vaccine itself and guarantee its effectiveness during vaccine testing and marketing. At the same time, in the publicity and education for FSWs in entertainment venues, more attention should be paid to objectively explaining the benefits of HPV vaccination in reducing the incidence of HPV infection and CC. In addition, they should enhance the concept that primary prevention measures such as vaccination are beneficial to individuals in controlling the development of diseases, so that the public can establish the awareness that prevention is more important than treatment, and gradually eliminate the occurrence of high-risk sexual behavior.

### Behavioral skill factors

The IMB model suggests that even if an individual has a high willingness and perception in behavioral change, certain behavioral skills need to be mastered before individual’s behavior can be changed [24]. This survey found that FSWs in entertainment venues with higher behavioral skills scores were more willing to receive HPV vaccination because they had good self-decision-making qualities and efficacy, and were able to communicate and exchange HPV vaccine-related matters with their relatives, friends and medical personnel. In this way they could accessed HPV vaccination institutions and procedures, and were willing to receive the HPV vaccine. The willingness of those who had mastered more behavioral skills of receiving HPV vaccine, i.e., self-decision-making and self-efficacy, was 1.5 times higher than those who could not receive HPV vaccine. Vaccination was 1.130 and 1.135 times more willing to be received those FSWs who could not grasp it, respectively. It is suggested that the future direction of popularization should appropriately add content that introduces the behavioral skills of HPV vaccination, such as the location of vaccination venues, booking methods and the vaccination process, so as to increase the willingness of FSWs in entertainment venues to receive HPV vaccination.

### Multifactorial analysis of HPV vaccination intentions

The results of the analysis showed that the willingness of FSWs in entertainment venues to be vaccinated against HPV was related to the class of the venue, smoking and alcohol habits, whether they agreed that the vaccine was effective in preventing diseases, whether they had heard of HPV vaccination, perceived benefits of HPV vaccination and the acquisition of more behavioral skills, i.e., self-decision-making and self-efficacy, to be vaccinated against HPV. Among them, the lower the grade of the venue, the more reluctant the FSWs were to be vaccinated against HPV, and the willingness to be vaccinated was 0.693 times higher than that of the previous grade for each stepwise decrease in the grade. This may be due to the fact that FSWs in high-end entertainment venues were required to go through some agreed criteria for selection as well as pre-service training before taking up their jobs, and that they were more aware of HPV and other sexually related infectious diseases and their understanding of the means to prevent them. This increased their awareness of sexual safety making them more profitable in the sex trade industry. The lack of management and training for FSWs in medium-grade and low-grade entertainment venues, and their lack of awareness of self-protection and acceptance of sex-related education and training in particular, suggested that local supervisory authorities should register FSWs in these establishments. They should also carry out health education and training on a regular basis, so as to enable them to protect their own health and at the same time as to reduce the spread of sex-related diseases at the source of the problem.

In conjunction with the actual situation of outreach interventions for FSWs in entertainment venues by the Centre for Disease Control and Prevention in an urban area of Guangxi, China, and other agencies, it has been shown that face-to-face, in-depth exchanges with FSWs are still difficult. For example, some publicity and education are only allowed in the lobby, and in some venues, only the foreman receives them. Because of the risk of being cracked down on by public security authorities, FSWs in entertainment venues, especially those in low-grade venues, are very wary of communicating with investigators and are evasive of survey questions. This undoubtedly makes it even more difficult to popularize and promote the vaccination of HPV and the HPV vaccine. In addition, due to the special nature of the investigation sites and the lack of intervention funds, the enthusiasm of grass-root staff is difficult to mobilize, and some departments are understaffed. Therefore, it can difficult to carry out the relevant work, so that the outreach intervention programs will inevitably become a mere formality. This suggests that the intervention mode of disease control and community health centers needs to be changed in the future, and that national government departments should increase funding, while at the same time introducing relevant policies and regulations, standardizing the management of entertainment venues where commercial sex transactions take place. They should also provide practical assistance to FSWs in entertainment venues in the areas of disease detection and mental health regulation, in order to strengthen the intervention of sexually transmitted infections, and to increase the rate of HPV vaccination.

This study has several strengths. Firstly, this study paid more attention to FSWs in entertainment venues, as there were fewer studies related to HPV vaccination in this population. Secondly, this study designed a survey questionnaire based on the IMB model to analyze the effects of information, motivation and behavioral skills on the willingness to receive HPV vaccination, and it was able to comprehensively analyze the factors influencing the willingness to receive this among FSWs in entertainment venues in an urban area of Guangxi, China. However, this study has some limitations. Because it was a cross-sectional study, it was not possible to provide inferential causality. This study used a purposeful sampling technique, and the findings may be subject to selection bias. Moreover, the questionnaires were filled out by the respondents themselves, and the information collected involved sensitive information as it related to personal sexual behavior and history of cancer among friends and relatives. It is possible that the respondents might provide false information to these types of enquiry and the findings may be subjected to information bias. It should also be noted that the factors found in this study to influence HPV vaccination intentions may not be nationally representative, and therefore they need to be interpreted with caution.

## Conclusions

In summary, FSWs in entertainment venues in an urban area of Guangxi, China, had a higher willingness to be vaccinated against HPV, and this desire was associated with the class of the venue as well as their tobacco and alcohol addiction in the basic profiles of the study population. Based on the information, motivation and behavioral skills aspects of the IMB model, whether one agrees that the vaccine is effective in preventing disease, whether one has heard of the HPV vaccine, perceived benefits of HPV vaccination and acquiring more behavioral skills of HPV vaccination i.e., self-decision making and self-efficacy were the influencing factors affecting the willingness of the research subjects to be vaccinated. Local governments and preventive healthcare institutions should strengthen the management of FSWs in entertainment venues, avoiding high-risk sexual behavior and effectively safeguarding the physical and mental health of these women. At the same time, they should carry out wide-ranging publicity campaigns and education on HPV and other sexually transmitted diseases, innovate the ways and forms of science popularization and focus on introducing the benefits of HPV vaccine and other primary prevention measures for individuals. In addition, they must also study the behavioral skills for HPV vaccination, so as to increase the willingness of FSWs to be vaccinated against HPV vaccine as well as the vaccination rate. They should also push forward the process of the National Immunization Program in order to promote the construction of a healthier China.

## Data Availability

The datasets used and/or analyzed during the current study available from the corresponding author on reasonable request.
